# Polymer‐Grafted Gold Colloids and Supracolloids: From Mechanisms of Formation to Dynamic Soft Matter

**DOI:** 10.1002/marc.202400851

**Published:** 2025-01-09

**Authors:** Christian Rossner

**Affiliations:** ^1^ Leibniz‐Institut für Polymerforschung Dresden e.V. Hohe Straße 6 D‐01069 Dresden Germany; ^2^ Faculty of Chemistry and Food Chemistry Technische Universität Dresden D‐01069 Dresden Germany; ^3^ Department of Polymers University of Chemistry and Technology Prague Technická 5 Prague 6 166 28 Czech Republic

**Keywords:** end‐grafted polymers, gold nanoparticles, hybrid nanomaterials, nanostructure formation, responsive nanomaterials, self‐assembly, supracolloids

## Abstract

Gold nanoparticles represent nanosized colloidal entities with high relevance for both basic and applied research. When gold nanoparticles are functionalized with polymer‐molecule ligands, hybrid nanoparticles emerge whose interactions with the environment are controlled by the polymer coating layer: Colloidal stability and structure formation on the single particle level as well as at the supracolloidal scale can be enabled and engineered by tailoring the composition and architecture of this polymer coating. These possibilities in controlling structure formation may lead to synergistic and/or emergent functional properties of such hybrid colloidal systems. Eventually, the responsivity of the polymer coating to external triggers also enables the formation of hybrid supracolloidal systems with specific dynamic properties. This review provides an overview of fundamentals and recent developments in this vibrant domain of materials science.

## Graphical abstract

The role of polymer molecules as end‐grafted ligands at gold nanoparticles in engineering nanoparticle structure, functionality, and arrangement into higher‐order supracolloidal structures is reviewed. Synthesis, characterization, underlying mechanisms of structure formation as well as possibilities of dynamic polymer/gold supracolloids are discussed.

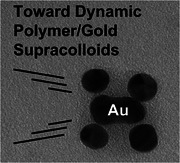

## 1 Background

Inorganic nanoparticles (NPs) grafted with polymer molecule ligands combine distinct properties in a single, nanosized entity:^[^ ^1^ ^]^ Depending on its chemical composition the inorganic particle may provide plasmonic,^[^ ^2^ ^]^ excitonic,^[^ ^3^ ^]^ or magnetic functionality,^[^ ^4^ ^]^ while the polymer ligand shell contributes soft properties and additional functionality.^[^ ^5^ ^]^ The polymeric coating also determines the interactive behavior of these nanoparticles, like for example responsivity toward external stimuli.^[^ ^6^ ^]^ Thus, the scope of inorganic nanoparticles, highlighted, e.g., by the 2023 Nobel Prize in Chemistry, can be widened significantly by the possibilities opened up by a polymer coating.

**Figure 1:**
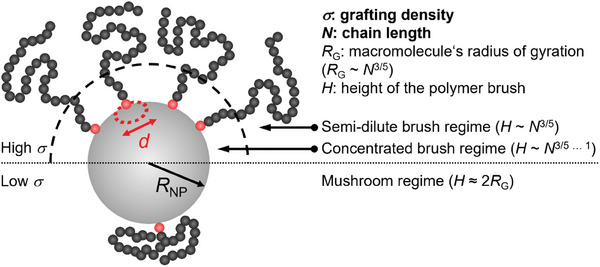
Illustration of chain conformations and resulting polymer layer height, *H*, for polymer‐grafted nanoparticles and their dependence on chain length, *N*, and grafting density, σ. The surface‐grafted polymer molecules occupy a circular footprint area with diameter *d*.

In an idealized, illustrative view, the grafted polymer layer is made from monodisperse linear polymer molecules that are grafted to the nanoparticle surface by an end monomer with surface affinity. When the polymeric architecture of grafted macromolecules is fixed in that manner, the shell architecture is governed by two important quantities: Polymer molar mass, which is proportional to the number of repeat units, *N*, and grafting density defined as the number of surface‐anchored polymer molecules per surface area, σ. The influence of *N* and σ on the shell architecture can be understood in terms of scaling arguments formulated first for star‐shaped polymers;^[^ ^7^ ^]^ and later extended to polymer molecules anchored to a core with convex curvature.^[^ ^8^ ^]^ Few assumptions like the equal stretching approximation lead to the following scaling laws. For low enough grafting density, polymer conformations are not perturbed by neighboring grafted molecules, and thus the polymer height, *H*, is roughly equal to the dimension of free polymer molecules, 2*R*_G_. (With *R*_G_ scaling as *N* ^3/5^ for an athermal solvent). A so‐called footprint area can be assigned to the grafted polymer molecules,^[^ ^9^, ^10^ ^]^ defined as a circular area with a diameter *d* equal to the distance between neighboring grafting points, i.e.: (π d^2^)/4. When σ increases beyond the point where this footprint diameter *d* < 2*R*_G_, interactions with neighboring surface‐grafted macromolecules invoke a stretching, resulting in a larger scaling exponent, *N* ^3/5…1^ (depending on surface curvature), as is also found experimentally.^[^ ^11^ ^]^ This condition is sometimes referred to as the concentrated polymer brush regime. The increased scaling exponent only persists until a certain distance from the core beyond which interactions between macromolecules are relaxed, due to the convex confinement geometry. Beyond that distance, chain conformations are less perturbed, and the initial *N* ^3/5^ scaling of layer height is restored. This condition can be referred to as the semi‐dilute polymer brush regime. These relationships are schematically illustrated in **Figure** **1**.

The conformational degrees of freedom of the brush layer under good solvency conditions prevent nanoparticles from associating due to van der Waals attraction, a phenomenon called steric stabilization. This can be discriminated from electrostatic stabilization which is typically employed to stabilize colloidal particles that do not carry a polymer ligand shell. Such electrostatically stabilized colloids aggregate in electrolyte solutions (by charge screening and concomitant destabilization), which can be prevented by a polymer brush coating.^[^ ^12^ ^]^ For a comprehensive discussion of the relevant interaction potentials and their effect on colloidal stability, the reader is referred to a recent review.^[^ ^13^ ^]^ Beyond colloidal stability, the scaling of polymer layer height can be exploited to synthesize core−shell‐type nanoparticles with defined polymer ligand shell,^[^ ^14^ ^]^ as will be discussed in more detail in Section 2.1. At this point, the importance of *N* and σ for the behavior of polymer‐grafted nanoparticles is exemplified by the following: i) Below a certain threshold value of σ, surface‐pinned micelles may form under reduced solvency conditions for the polymer layer; the number of these pinned micelles depends on *N*.^[^ ^15^ ^]^ ii) The morphology of self‐assembled structures derived from polymer‐grafted nanoparticles depends on *N*.^[^ ^16^ ^]^ iii) Chain length and grafting density determine the dispersion state of polymer‐grafted nanoparticles within a polymer matrix. Dispersion is favored for high σ and *N* values, whereas segregation into 1D, 2D, and eventually globular nanoparticle aggregates within the matrix is observed upon gradually decreasing these values.^[^ ^17^ ^]^ iv) The toughness of bulk material made of polymer‐grafted nanoparticles increases with increasing *N*, approaching the toughness value for pure high molar mass bulk polymer.^[^ ^18^ ^]^

To respond to the demand of controlling the architecture of a “hairy” coating layer, synthetic methodologies for functionalizing gold nanoparticles with end‐grafted polymer molecules have been developed and refined during the last decades. The presented discussion will introduce these methodologies and discuss underlying mechanistic principles (Section 2.1) followed by an overview of analytical approaches for characterizing these systems (Section 2.2). Next, the possibilities that are offered by existing synthetic approaches for controlling structural and functional complexity at the single‐particle level will be explored (Sections 2.3 and 2.4). The polymeric coating layer can also be used to engineer interactions between different nanoparticles, thereby enabling the self‐assembly or directed assembly of nanoparticles into supracolloidal species in solution (as will be discussed in Section 3). A range of structurally well‐defined supracolloidal structures has been accessed recently, featuring unique e.g. optical properties. A further option in creating and harnessing useful functional supracolloidal properties arises from controlling their dynamic properties, which is demonstrated by several recent examples that will be introduced toward the end of this article (Section 4).

## 2 Polymer‐Grafted Gold Nanoparticles

Because of their chemical stability and the relative ease of surface modification, gold nanoparticles have become one of the most studied classes of inorganic nanomaterials. A huge part of their appeal can be attributed to their special optical properties, which result from the resonant excitation of the so‐called plasmon resonance by light of visible or NIR frequencies, a collective oscillation of valence electrons confined to the particle.^[^ ^19^ ^]^ The spectral position and magnitude of this resonance are strongly influenced by the gold nanoparticle's dimension and shape. Today, wet‐chemical synthesis methods are mature enough to map these effects systematically: The size‐selective synthesis of gold nanospheres,^[^ ^20^, ^21^ ^]^ nanocubes,^[^ ^22^ ^]^ nanorods,^[^ ^23^ ^]^ (and other more sophisticated, e.g., chiral shapes)^[^ ^24^, ^25^ ^]^ allows to adjust the optical properties of gold nanoparticles. Fine‐tuning these properties for a given nanoparticle dimension and shape becomes possible by adapting the dielectric environment, which can be done by adjusting the chemical composition and thickness of a polymeric coating layer.^[^ ^26^ ^]^ The sensitivity to the dielectric environment makes polymer‐grafted nanoparticles to nanoscale sensors, which can be used for analyte detection using appropriate optics for the read‐out.^[^ ^27^, ^28^ ^]^ Having said that, it should be mentioned that the application potential of polymer‐coated gold nanoparticles is not limited to sensing but spans a wide scope. A comprehensive discussion can be found in recent reviews dedicated to their applications in particular domains, such as, e.g., catalysis,^[^ ^29^ ^]^ nanomedicine,^[^ ^30^ ^]^ and optics,^[^ ^31^ ^]^ to name just a few.

In the following section, synthetic approaches for controlling the polymer layer architecture on gold nanoparticles are categorized and discussed in comparison. This will be followed by a discussion of characterization methods for counting the number of surface‐attached polymer molecules. The second part of this section explores the versatility of hairy gold nanoparticles in terms of structure and functionality.

### 2.1 Synthetic Access to Polymer‐Grafted Gold Nanoparticles

**Figure 2:**
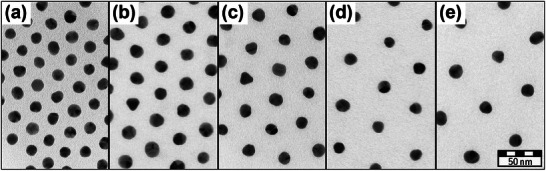
Self‐assembled monolayers of gold nanoparticles grafted with linear polymers of *N*‐isopropylacrylamide with increasing molar masses: a) 14.9 kDa; b) 25.8 kDa; c) 37.9 kDa; d) 47.4 kDa; e) 74.4 kDa. Adapted with permission from.^[^ ^14^ ^]^ Copyright 2013, American Chemical Society.

Two strategies for accessing gold nanoparticles with a grafted polymer layer can be discriminated. In the so‐called in situ strategy, a gold salt precursor is reduced under the presence of polymer molecules with gold‐affine end monomer in a one‐pot reaction.^[^ ^32^ ^]^ While this is a very straightforward approach, it typically leads to comparably small gold nanoparticles with relatively large size dispersity. Despite progress in this domain, this strategy is used mostly in cases of very reactive end groups on the applied polymer ligands^[^ ^33^ ^]^ or because of other restrictions. To make use of the huge variety of well‐defined gold nanoparticles mentioned above, the so‐called ex situ strategy is typically pursued, in which nanoparticle synthesis and functionalization with polymer grafts are separated. Within the frame of this strategy, it is possible to synthesize well‐defined core−shell‐type nanoparticles with defined shell dimensions, as depicted in **Figure** **2**.^[^ ^14^ ^]^ In the transmission electron micrographs shown in Figure 2, only the gold cores are directly visible, but the polymer shell keeps these cores at defined and uniform distances within self‐assembled 2D patterns.

**Figure 3:**
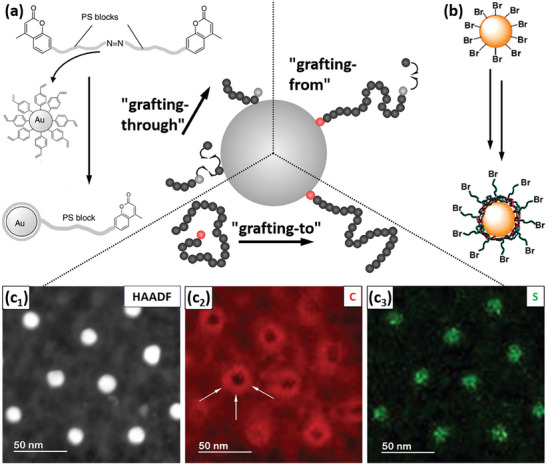
Overview of synthetic approaches for functionalizing pre‐synthesized gold nanoparticles with polymer molecule ligands. a) The grafting‐through strategy, in which the active chain end of a growing macromolecule propagates “through” surface‐immobilized monomer species. Scheme adapted with permission.^[^ ^38^ ^]^ Copyright 2011, John Wiley and Sons. b) The grafting‐from strategy, in which a surface‐immobilized initiator or chain‐transfer agent initiates macromolecular chain growth, such that macromolecular species grow away from the surface. Scheme adapted with permission.^[^ ^40^ ^]^ Copyright 2008, American Chemical Society. c) The grafting‐to strategy, in which pre‐synthesized polymer molecules with surface‐affine groups (typically end monomers) are attached to the surface. c_1_) High‐angle annular dark‐field image. c_2_) Carbon map. c_3_) sulfur map. Adapted with permission.^[^ ^44^ ^]^ Copyright 2016, John Wiley and Sons.

The polymeric grafts can be installed by three different approaches, which can be categorized according to the surface‐immobilized species involved (**Figure** **3**).

In the grafting‐through approach, a growing active species attaches to a surface‐immobilized monomer, which then becomes the active chain end from which chain propagation progresses (Figure 3a). When combined with controlled polymerization techniques such as Reversible Addition−Fragmentation chain Transfer (RAFT) polymerization, this way of anchoring macromolecules to solid inorganic particles was employed successfully in the exfoliation of clay (montmorillonite) nanosheets.^[^ ^34^, ^35^ ^]^ In combination with gold colloids, the grafting‐through approach has been used successfully,^[^ ^36^ ^]^ although it is employed less frequently. In some of the few examples, the grafting‐through approach was suggested as a means of gold‐nanoparticle monofunctionalization, which would occur based on the full consumption of all initially present, surface‐bound monomers after initiation with one initiator fragment.^[^ ^37^, ^38^ ^]^

The grafting‐from approach is characterized by initiator/chain‐transfer molecules being initially immobilized at the nanoparticle surface, which then results in active macromolecular chains that grow away from the surface (Figure 3b). This has important implications for the formation of macromolecules by this approach, which, e.g. for radical polymerization, differs from the bulk solution polymerization scenario: The surface‐immobilization of these active chains was shown^[^ ^39^ ^]^ to lead to lowered and chain‐length independent termination rate coefficients, by suppressing diffusion‐controlled termination pathways, resulting in termination rate coefficients that are governed by reaction‐diffusion. Besides these peculiarities, the use of a controlled polymerization technique offers additional options for the macromolecular design of the grafted polymer layer on gold nanoparticles: Due to the living nature of, e.g., a surface‐initiated atom‐transfer radical polymerization (ATRP), one may choose to synthesize a first, nanoparticle‐adjacent polymer block with some degree of cross‐linking; and another, nanoparticle‐remote non‐cross‐linked block.^[^ ^40^ ^]^ This combines the colloidal stability provided by the outer block with increased thermal stability as a consequence of the cross‐linking in the inner block.

**Figure 4:**
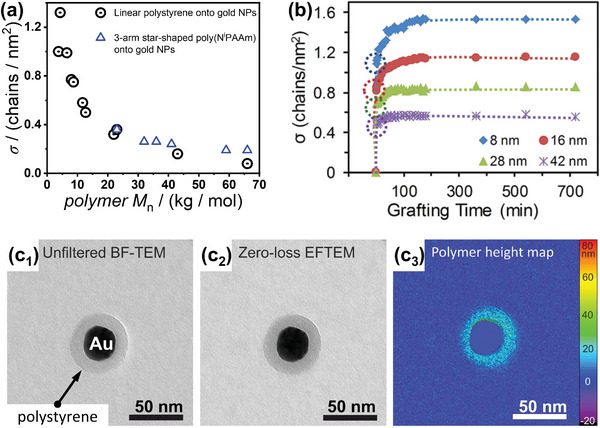
a) Results from thermogravimetric analysis showing decreasing grafting density for increasing polymer molar mass resulting from a grafting‐to approach at spherical gold nanoparticles (for two different systems: black data points:^[^ ^9^ ^]^ linear, thiol‐terminated polystyrene ligands grafted onto 11 nm citrate‐reduced gold nanoparticles; blue data:^[^ ^66^ ^]^ 3‐arm star‐shaped, trithiocarbonate‐terminated poly(*N*‐isopropylacrylamide) ligands grafted onto 13 nm citrate‐reduced gold nanoparticles. b) Results from NMR‐based characterization for the grafting of PEG ligands onto spherical gold nanoparticles of distinct sizes, reveal the effect of surface curvature on achievable grafting densities. Reproduced,^[^ ^61^ ^]^ Creative Commons under CC BY‐NC 3.0. Determination of polymer‐grafting density at the single‐particle level by energy‐filtered TEM: c_1_) Conventional (unfiltered) bright‐field TEM image; c_2_) Zero‐loss filtered bright‐field TEM image; c_3_) Polymer height map based on the Poisson ratio of the former two images. Adapted,^[^ ^62^ ^]^ Creative Commons under CC‐BY‐NC‐ND 4.0.

In the majority of cases, however, polymers are immobilized onto gold nanoparticles following the grafting‐to approach (Figure 3c). A prerequisite for this is the incorporation of functional groups with affinity to the gold surface. Typically these are end monomers, but for some cases, one^[^ ^41^ ^]^ or more^[^ ^14^ ^]^ of such groups can be also incorporated into the polymeric main chain (leading to V‐shaped or Guiselin‐type brushes, respectively). The gold‐affine moieties are typically chosen as sulfur‐containing functional groups, which can take the form of thiols,^[^ ^9^ ^]^ dithioesters,^[^ ^42^ ^]^ or trithiocarbonates,^[^ ^14^, ^43^, ^44^ ^]^ but also other interactions like halogen bonds to gold^[^ ^45^ ^]^ or binding via phenylacetylenes^[^ ^46^ ^]^ and *N*‐heterocyclic carbenes have been suggested.^[^ ^47^, ^48^, ^49^ ^]^ Unlike the adsorption of small molecules onto nanoparticle surfaces which typically approaches thermodynamic equilibrium,^[^ ^50^ ^]^ the grafting‐to of macromolecular species is a barrier‐limited process, during which free‐energy barriers dynamically build up:^[^ ^51^ ^]^ At early stages of the grafting process, these barriers result from the conformational degrees of freedom of the macromolecules that become immobilized; at later stages, already surface‐attached macromolecules add to the free‐energy barrier. In line with this barrier‐controlled polymer grafting, higher grafting densities are experimentally observed when the grafting‐to is performed at elevated temperatures^[^ ^52^ ^]^ or under sonication.^[^ ^53^ ^]^ As these barriers depend on the chain length of the grafting macromolecules, the grafting of shorter chains is kinetically favored.^[^ ^51^ ^]^ For synthetic polymers (with a dispersion with respect to chain length), this results in a corresponding chain‐length bias between the polymer molecule ensemble before surface grafting and the eventually surface‐immobilized macromolecules.^[^ ^54^, ^55^ ^]^ It was shown that solvency conditions of the grafting polymers have a strong influence on the preferential surface grafting of shorter chains, and the effect is more pronounced for good solvency conditions compared with close‐to‐θ conditions.^[^ ^55^ ^]^ In general, low molar‐mass dispersity and high chain‐end fidelity^[^ ^56^ ^]^ are sought to minimize this chain‐length bias, which is why further developments of controlled polymerization techniques, e.g. photo‐iniferter RAFT polymerization,^[^ ^57^ ^]^ are highly relevant in the context of producing well‐defined polymer ligands as gold nanoparticle coatings.^[^ ^58^ ^]^ The influence of chain length on the surface grafting propensity also results in a trend of decreasing grafting densities with increasing molar mass of grafting polymers, and a limitation concerning accessible grafting densities in general (**Figure** **4**). However, for many purposes the achievable grafting densities resulting from the grafting‐to approach are sufficient, and as we will see, for some purposes grafting densities are even intentionally lowered, which can be achieved by adjusting the ratio between the number of macromolecules offered in the grafting‐to and available surface area. This ratio is sometimes referred to as target grafting density. It can be adjusted experimentally based on total gold content (in many cases accessible via extinction spectroscopy)^[^ ^59^ ^]^ and gold nanoparticle dimension and shape (as well as dispersity concerning these properties). By definition, the target grafting density during the grafting‐to provides an upper limit for the actual grafting density after completion of the grafting process. Thus, if this information is required, the actual grafting density has to be determined for synthesized polymer‐grafted nanoparticles. This brings us to the next section, where experimental techniques for quantifying polymer grafting at nanoparticles are discussed.

### 2.2 Quantifying Polymer Ligand Grafting at Gold Nanoparticles

While the general success of forming a grafted polymer layer at gold nanoparticles can be judged relatively easily (e.g., by visible/NIR extinction spectroscopy and dynamic light scattering), quantitative characterization can be challenging. This is particularly true for the determination of the grafting density. The most commonly used method to determine this quantity still is thermogravimetric analysis. If the structural identity of gold nanoparticles and the molar mass of the polymer ligands are known, the weight retention during such measurements (assumed to be equal to gold content) can be related to the grafting density. Results obtained in that manner are shown in Figure 4a. Although thermogravimetry yields reasonable results, it requires comparably high amounts of nanoparticles (typically a few milligrams), which can be problematic in cases where the nanoparticle synthesis is tedious, like for non‐spherical gold‐nanoparticle shapes. Therefore, non‐destructive methods for accessing grafting density values or methods requiring only a minimal amount of material are in great demand. Many of these methods and their advantages/disadvantages are summarized and comprehensively discussed in a review article,^[^ ^60^ ^]^ while some more recent developments^[^ ^61^, ^62^ ^]^ highlight the abiding need for widened analytical possibilities.

Spectroscopic techniques can offer these possibilities, while in some cases additional labeling is required. For example, if the polymer ligands provide end groups that can be conjugated to a dye, extinction or fluorescence spectroscopy can be used to quantify grafting densities.^[^ ^63^, ^64^ ^]^ However, care has to be taken since the presence of gold nanoparticles can perturb the spectroscopic signals in a distance‐dependent manner. To avoid such extinction/fluorescence modulation leads to false quantitative results, spectroscopy can be performed for non‐grafted, excess polymer molecules in the supernatant (indirect method). These excess polymer molecules can be detected using colorimetric assays such as Ellman's reagent for the detection of thiols.^[^ ^63^, ^65^ ^]^ Another possibility is the etching of the gold core, which releases the surface‐bound macromolecules (direct method).^[^ ^64^ ^]^ Generally, these fluorescence/extinction spectroscopy methods involve additional labeling steps which may introduce additional sources of experimental error; and for direct determination the method is again destructive, typically using cyanide etching, which should be avoided if possible. Recently, an approach^[^ ^61^ ^]^ for label‐free determination of polymer grafting densities at gold nanoparticles was developed, which is based on the broadening of the proton nuclear magnetic resonance upon surface grafting. This method allowed to track the surface grafting of thiol‐terminated poly(ethylene glycol) (PEG) ligands to gold nanoparticles in a time‐resolved manner. As these spectroscopy‐based methods enable grafting‐density determination more conveniently compared with TGA, these were used to investigate what factors influence σ values and the corresponding trends. In addition to the established dependence on the molar mass of the grafting polymer (Figure 4a), a dependence on the dimension of spherical gold nanoparticles (i.e., their surface curvature) is revealed, where increased grafting densities are observed for smaller nanoparticles (Figure 4b).^[^ ^61^ ^]^ Resulting polymer grafting densities are also strongly influenced by the kind of initially present small‐molecule ligands that stabilize the gold nanoparticles before polymer grafting. Here, higher grafting densities are observed for initially citrate‐stabilized gold nanoparticles compared with surfactant‐stabilized (surfactants being cetyltrimethylammonium bromide (CTAB)^[^ ^61^ ^]^ and ‐chloride (CTAC))^[^ ^64^ ^]^ gold nanoparticles.

Until recently, all available methods for measuring polymer grafting densities at nanoparticles were ensemble‐based methods, which produce average values and are not capable of determining polymer load at the single nanoparticle level or distinct sites of anisotropic nanoparticles. We tackled this challenge by demonstrating the capability of energy‐filtered transmission electron microscopy (EFTEM) to image and discriminate the inorganic core and its polymer coating and to determine the polymer shell volume (Figure 4c).^[^ ^62^ ^]^ This approach also enables the site‐selective quantification of polymer load at anisotropic nanoparticles, as will be discussed later in this review.

### 2.3 Structural Complexity at the Single‐Particle Level

The available structural complexity in polymer‐grafted nanoparticles can be drastically increased by introducing heterogeneities in the surface coating. This can be achieved based on the “soft” properties of the polymer ligand layer, which imparts these systems with the required structural plasticity.^[^ ^67^ ^]^ Such heterogeneities break the symmetry of formed colloidal species and thereby strongly influence their behavior toward biological and non‐biological entities.^[^ ^68^ ^]^

**Figure 5:**
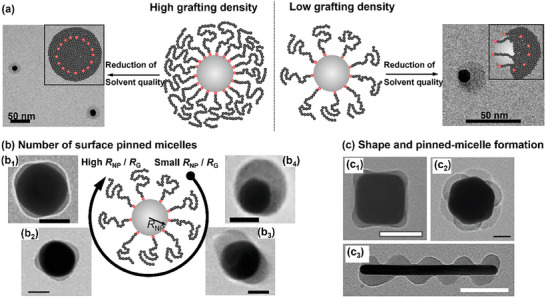
a) Cryogenic transmission electron microscopy images of poly(*N*‐isopropylacrylamide)‐grafted gold nanoparticles. The images were both captured after plunge freezing from poor solvency conditions (above the polymer's lower critical solution temperature in water). Distinct polymer‐shell morphologies were observed as a consequence of different grafting densities. Adapted with permission.^[^ ^70^ ^]^ Copyright 2018, American Chemical Society. b) Transmission electron microscopy images of polystyrene‐grafted gold nanoparticles after drop‐casting from poor solvency conditions. The number of surface‐pinned “octopus”‐micelles is governed by the molar mass of the polystyrene ligands and the dimension of the gold nanoparticle. Scale bars are: (b_1_) 50 nm; (b_2_) 20 nm; (b_3_) 25 nm; (b_4_) 20 nm. Adapted with permission.^[^ ^15^ ^]^ Copyright 2016, Springer Nature. c) Transmission electron microscopy images showing examples of “octopus”‐micelle morphologies at shaped gold nanoparticles: (c_1_) Gold nanocubes, scale bar is 50 nm. Adapted with permission.^[^ ^72^ ^]^ Copyright 2017, American Chemical Society. (c_2_) Gold nanooctahedra, scale bar is 20 nm. Adapted with permission.^[^ ^74^ ^]^ Copyright 2018, John Wiley and Sons. (c_3_) Gold nanorods, scale bar is 100 nm. Adapted with permission.^[^ ^75^ ^]^ Copyright 2019, John Wiley and Sons.

In grafted homopolymer layers on gold nanoparticles, structural heterogeneities can result from a process called constrained surface de‐wetting, which is a phenomenon known from sparsely grafted polymer brushes at flat surfaces.^[^ ^69^ ^]^ The group of Kumacheva recently presented a design paradigm for surface patterning of gold nanoparticles based on this de‐wetting mechanism: Below a certain threshold grafting density, polymer brushes segregate into “octopus”‐like micelles under poor solvency conditions for the polymer, where the minimization of the polymer‐solvent interfacial energy overcompensates the entropic penalty of stretching the “octopus's legs”.^[^ ^15^ ^]^ This transition can be revealed in the native solution environment by cryogenic transmission electron microscopy imaging (**Figure** **5**a).^[^ ^70^ ^]^ Plunge freezing of a colloidal solution containing poly(*N*‐isopropylacrylamide)‐grafted gold nanoparticles (15 nm) at 45 °C (i.e., above the polymer's lower critical solution temperature, thus poor solvency conditions) resulted in uniform polymer coating layers for high grafting densities; while partially de‐wetted, Janus‐type particles were observed for lower grafting densities. In such systems, the number of formed “octopus”‐micelles is determined by the ratio of nanoparticle diameter 2*R*_NP_ and the *R*_G_ of grafted polymers.^[^ ^15^ ^]^ For bigger particles or grafted polymer molecules with smaller *R*_G_, not all polymer molecules can merge into a single “octopus” micelle, and thus the number of “octopus” micelles increases with increasing *R*_NP_/*R*_G_ ratio (Figure 5b). At anisotropic nanoparticles, the “octopus” micelles typically form at high curvature sites, which can be rationalized by the theoretically expected^[^ ^71^ ^]^ higher polymer grafting densities at these loci, which in turn favor the polymer segregation at these positions.^[^ ^72^, ^73^ ^]^ In line with this argument, the preferred formation of “octopus” micelles at vertices and corners of polystyrene‐grafted gold nanocubes^[^ ^72^ ^]^ or octahedra^[^ ^74^ ^]^ is indeed the respective experimental observation (Figures 5c1,c2). Additionally, for gold nanorods of high aspect ratio, polystyrene ligand segregation into a helicoidal pattern is observed in some regions of the associated *N*/σ parameter space (Figure 5c3).^[^ ^75^ ^]^

**Figure 6:**
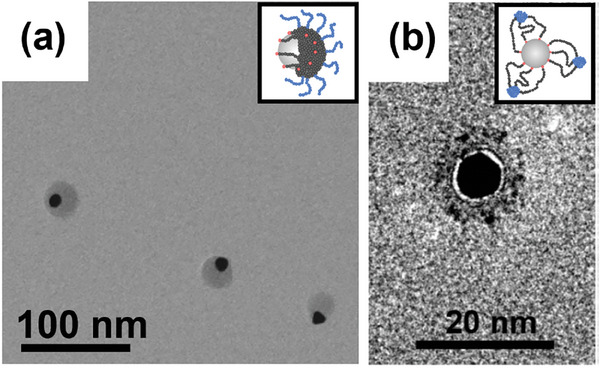
a) Transmission electron microscopy imaging of gold nanoparticles grafted with poly(styrene)‐*block*‐(4‐vinylbenzoic acid). The imaging was performed after drop casting at poor solvency conditions for the inner polystyrene block and good solvency conditions for the outer, nanoparticle‐remote poly(4‐vinylbenzoic acid) block. Adapted with permission.^[^ ^77^ ^]^ Copyright 2019, John Wiley and Sons. b) Transmission electron microscopy imaging of gold nanoparticles grafted with polystyrene‐*block*‐(methacrylic acid).^[^ ^82^ ^]^ The imaging was performed after drop casting at good solvency conditions for the inner polystyrene block and bad solvency conditions for the outer, nanoparticle‐remote poly(methacrylic acid) block, i.e., “flipped” solvency conditions compared with the scenario in (a).

While a rich phenomenology in surface patterns can be achieved based on constrained surface de‐wetting with homopolymer ligands, the poor solvency condition that drives this process renders the produced species inherently colloidally unstable, and secondary aggregation processes typically occur concomitantly with the formation of surface patterns.^[^ ^15^, ^70^ ^]^ A rationale to stabilize “octopus”‐micelles at gold nanoparticles is using surface‐grafted block copolymers with an inner, nanoparticle‐adjacent segregating block, and an outer, nanoparticle‐remote stabilizing block^[^ ^76^, ^77^ ^]^ – a strategy analogous to the stabilization of all‐polymer “patchy” nanoparticles formed from the self‐assembly of triblock terpolymers with a core‐forming A block (representing the intrinsically phase‐separated gold nanoparticle), a B block from which the patches form, and a stabilizing C block.^[^ ^78^, ^79^ ^]^ Block copolymers comprising inner polystyrene blocks and nanoparticle‐remote poly(4‐vinylbenzoic acid) blocks enabled the formation and stabilization of “octopus” micelles at gold nanoparticles under poor solvency conditions for the polystyrene block (**Figure** **6**a).^[^ ^77^ ^]^ These patchy nanoparticles represent colloidally stable entities, as evidenced by dynamic light scattering and transmission electron microscopy imaging.^[^ ^77^ ^]^ Another work^[^ ^80^ ^]^ employed thermal equilibration between polymer‐grafted triblock terpolymers and free polymer ligands, demonstrating – in addition to colloidal stability – control over patch number and distribution.

In principle, the segregated domains may also be formed by the nanoparticle‐remote block, while the inner, nanoparticle‐adjacent block would act as a spacer between these segregated domains and the nanoparticle core, giving rise to planet−satellite‐type structures. Such structures have been predicted by (self‐consistent field) simulations involving diblock copolymers grafted to a solid sphere.^[^ ^81^ ^]^ These predicted structures were recently verified in experiments studying poly(styrene)‐*block*‐poly(methacrylic acid) diblock copolymers grafted to gold nanospheres (22 nm). De‐protonation of the nanoparticle‐remote poly(methacrylic acid) block in an organic solvent environment led to the collapse of this block, resulting in spherical segregated domains, separated from the gold core by the polystyrene spacer (Figure 6b).^[^ ^82^ ^]^

Structural complexity can also be achieved by employing mixed brush layers, in which (at least) two distinct polymer species are grafted to the nanoparticle surface, differing in either chain length or chemical composition. In the former scenario, bimodal brushes at the nanoparticle surface can be formed, which has been explored first and most detailed for polymer brushes at silica nanoparticles, formed by successive surface‐initiated polymerizations.^[^ ^83^ ^]^ For gold nanoparticles, bimodal distributions of molar masses of surface‐grafted polymers have been achieved in a grafting‐to approach, thoroughly considering the different surface attachment propensity associated with the different molar masses discussed in Section 2.1. That is, an excess of the high *N* polymer is required if roughly equimolar surface coverage of both polymers is strived.^[^ ^51^ ^]^ In the mentioned work,^[^ ^51^ ^]^ the grafted polymeric species were (3‐arm) star polymers with trithiocarbonate end monomers. Their grafting led to a bimodal distribution of free, non‐surface‐bound end monomers in the shell, which can be used to attach additional functional entities at two separate, well‐defined separations from the golden core nanoparticle.^[^ ^51^ ^]^ Another scenario is mixed brushes constituting compositionally different polymer ligands. These can give rise to phase separation phenomena, which can be used for structuring purposes: On spherical gold nanoparticles, phase separation in mixed brushes of PEG/polystyrene^[^ ^84^ ^]^ and poly(methyl methacrylate)/polystyrene^[^ ^85^ ^]^ led to Janus‐like structures, as evidenced by overgrowth of the PEG domains with silica^[^ ^84^ ^]^ or upon direct spectrum image mapping based on scanning transmission electron microscopy and electron energy‐loss spectroscopy.^[^ ^85^ ^]^ In mixed PEG/polystyrene brushes on gold nanorods, different phase‐separated morphologies have been observed dependent on the PEG/polystyrene grafting ratio.^[^ ^86^ ^]^ Such compositionally different mixed polymer brushes also provide opportunities by combining one stimulus‐responsive and one functionality‐carrying brush, where the functionality can be “hidden” in one state by the other brush, and activated on‐demand by an external trigger that collapses the responsive brush and thereby exposes the functional brush.^[^ ^87^, ^88^, ^89^ ^]^ Functional complexity in the polymer layers is discussed in its own right in the following section.

### 2.4 Functional Complexity at the Single‐Particle Level

The polymer layer attached to gold nanoparticles can carry additional functional entities, which open up another dimension of complexity for single polymer‐grafted gold nanoparticles. Because of the rich possibilities offered by polymer synthesis tools, opportunities for functional complexity are vast. In the following, three illustrative examples are presented.

**Figure 7:**
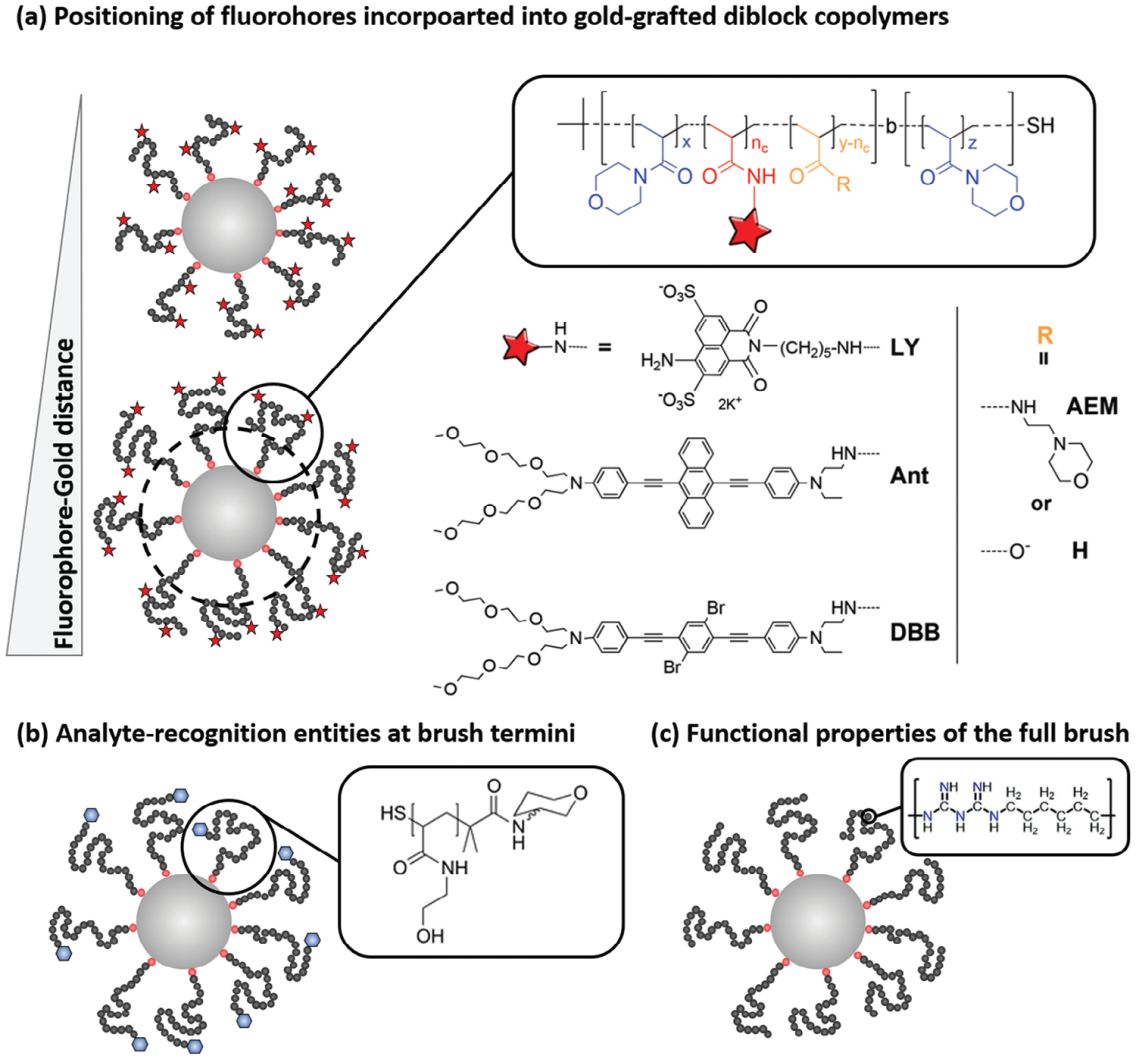
a) Incorporation of fluorophores into polymer brushes by co‐polymerization of (acrylamide‐type) monomer carrying different dye molecules. Poly(*N*‐acryloylmorpholine) homopolymer spacer blocks with adjusted chain length can serve to control the gold‐fluorophore separation. Chemical formulae adapted with permission.^[^ ^52^ ^]^ Copyright 2016, Royal Society of Chemistry. b) Incorporation of different 2‐deoxy‐2‐amino monosaccharides at nanoparticle‐remote termini of grafted polymer brushes. Chemical formula adapted with permission.^[^ ^96^ ^]^ Copyright 2014, American Chemical Society. c) Polymer ligand‐modified gold nanoparticles in which the polymer molecule as a whole possesses functional properties (antibacterial in this case.) Chemical formula adopted,^[^ ^98^ ^]^ Creative Commons under CC BY 4.0.

Incorporation of fluorophores into the polymer shell is tempting because fluorescence enhancement and quenching can be realized as a function of the distance between the fluorophores and the gold nanoparticle surface.^[^ ^90^ ^]^ This principle is already used in biosensing, where a pre‐quenched configuration can be turned into its luminescent form by a target biological entity, as demonstrated by in‐vitro measurements of the activity and inhibition of BACE1 (beta‐site Amyloid precursor protein‐cleaving enzyme 1, which is involved in the proteolytic formation of Amyloid‐beta peptides, their plaques representing a neuropathological correlate of Alzheimer's disease).^[^ ^91^ ^]^ Synthetically, fluorophores can, e.g., be integrated into surface‐immobilized polymer molecules by co‐polymerizing a fluorophore‐containing (acrylamide‐type) monomer with other (acrylamide/acrylate‐type) co‐monomers (**Figure** **7**a).^[^ ^52^ ^]^ Implementation of a block‐copolymer structure can serve to achieve a spacing between a fluorophore‐containing, nanoparticle‐remote block and a fluorophore‐free, nanoparticle‐adjacent spacer block. The distance‐dependent fluorescence‐intensity modulation could be demonstrated in these core−shell‐type hybrid nanoparticles.^[^ ^52^ ^]^ These nanohybrids also demonstrated potential for in‐vitro bioimaging.^[^ ^92^ ^]^

Moieties that can interact directly with (bio‐)analyte targets represent another category of functional groups in polymer brushes on gold nanoparticles. To facilitate interactions with the target molecules, these functional groups are often incorporated at the nanoparticle‐remote brush termini (Figure 7b). For example, these can be sugar moieties, which interact specifically with lectins: The presence of the target lectin can lead to particle association, as the complementary lectin can bridge between different nanoparticles, which is associated with a change of solution color due to plasmonic coupling effects.^[^ ^93^ ^]^ This sensing modality can be used to detect and discriminate different lectins.^[^ ^94^ ^]^ By changing the analyte‐detection moiety it is also possible to detect other bio‐analytes such as pathogens.^[^ ^95^ ^]^ The role of the polymer segment between the gold nanoparticle and the analyte‐binding entity is to ensure colloidal stability of the initial gold/polymer core−shell‐type nanoparticles, which can be compromised in the case of too short polymer segments.^[^ ^96^ ^]^ On the other hand, for too long polymer segments the plasmonic couplings upon analyte‐triggered nanoparticle agglomeration are weakened, and so is the optical read‐out. There is thus an optimized polymer length of the linking polymer segment for maximized sensing performance.^[^ ^96^ ^]^ Besides the length of the polymer segment, other properties like the chemical nature^[^ ^97^ ^]^ of the linking polymer also affect the magnitude of the response upon analyte binding. Thus, in many cases, the sensor performance can be optimized by modifying the design of the functionally passive polymeric linker component, which links the analyte‐recognition element and the gold nanoparticles (which provide the sensing capability).

In some cases, the whole grafted brush displays biological activity (Figure 7c), as is for example the case for antimicrobial polymers end‐grafted to gold nanoparticles.^[^ ^98^ ^]^ This particular combination merges the bacteria‐combatting properties of the polymer with the photothermal activity of gold nanoparticles (via plasmonic heating),^[^ ^99^, ^100^ ^]^ which can provide an additional lever for killing bacteria.^[^ ^98^ ^]^

## 3 Polymer‐Grafted Gold Supracolloids–Selected Examples

Polymer‐grafted gold nanoparticles introduced above form the building blocks for creating supracolloidal assembly structures. Formation of such assembly structures is mostly motivated by creating plasmon‐plasmon coupling effects between individual gold nanoparticles; or by synergistically combining the plasmonic properties of the gold nanoparticles with other dielectric, magnetic, or excitonic nanoparticles. Because the assembly state determines the presence and magnitude of these coupling and/or synergistic effects, nanoparticle placement inside the supracolloids is critical. This has motivated researchers to respond to this intellectual and application‐driven challenge of nanoparticle placement within supracolloids. Many of the developed solutions in this domain are based on specially designed surface‐grafted polymer ligands and the understanding of the specific colloidal interactions involved in these systems. While the literature on such supracolloidal systems is vast, only selected recent examples are discussed below, stepping up in complexity at the supracolloidal scale. The discussion starts with homo‐assemblies of spherical, polymer‐grafted gold nanoparticles in Section 3.1. In Section 3.2, homo‐assemblies of anisotropic polymer‐grafted gold nanoparticles are discussed, in which site‐selective interactions can play a role in determining supracolloidal structure. Co‐assemblies that include polymer‐decorated gold nanoparticles and other types of particles are discussed in Section 3.3. Here, the second type of nanoparticle differs in at least one of the following qualities: dimension, shape, or chemical composition.

### 3.1 Controlled Aggregation of Polymer‐Grafted Spherical Gold Nanoparticles

The assembly of polymer‐coated gold nanoparticles into a range of aggregate structures (incl. dimers, oligomers, chain‐like assemblies, and globular assemblies) can be promoted by surface‐grafted polymers. This research is in part driven by achievable material properties and potential applications that become possible in these plasmonically coupled gold nanoparticles. The most prominent example is probably given by plasmon rulers, which can be employed for distance determination in coupled gold‐nanoparticle pairs up to several tens of nm.^[^ ^101^ ^]^ Chain‐like assemblies of gold nanoparticles have been shown – upon incorporation into a polymer matrix–to serve as sensitive colorimetric stress sensors, based on their pronounced mechanochromic properties.^[^ ^102^ ^]^ More compact assemblies of gold nanoparticles, supporting many local hot‐spot sites, have been employed as surface‐enhanced Raman scattering (SERS) probes, where the SERS intensity depends on the assembly state. For example, poly(allylamine hydrochloride)‐coated gold nanoparticles were found to aggregate/dis‐assemble in a pH‐dependent manner, leading to SERS intensity increase/decrease of adsorbed molecular SERS tags.^[^ ^103^ ^]^ Concerning the mechanism of formation, it is possible to discriminate between unspecific interactions such as, e.g., solvophobic interactions that drive the assembly on one hand; and directed linking of nanoparticles through covalent or non‐covalent interactions promoted by surface‐grafted polymer species on the other hand.

**Figure 8:**
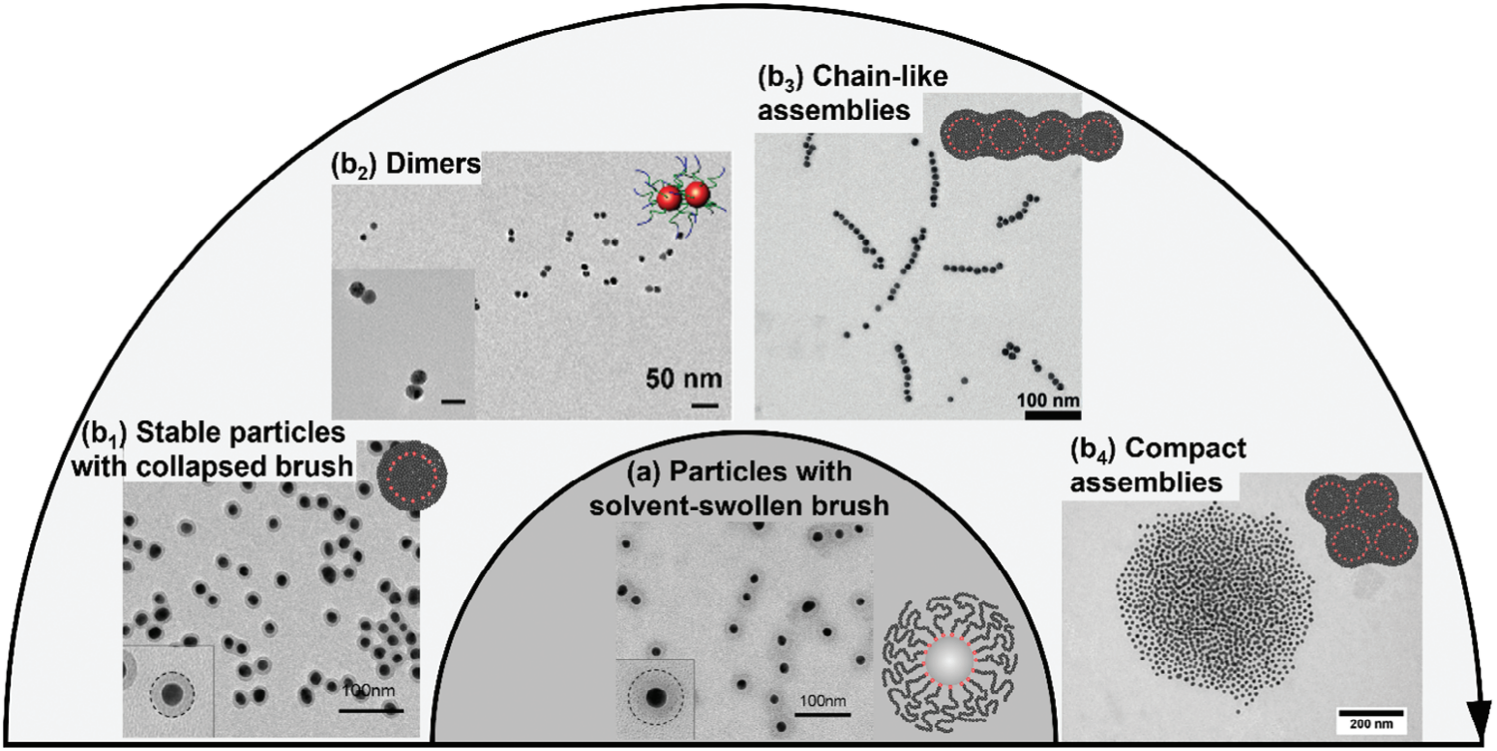
Observed structures after transitioning from good solvency conditions for a polymer layer at spherical gold nanoparticles (bottom center, dark gray background) to poor solvency conditions (light gray background). The balance between attractive (solvophobic) interactions and repulsive (mostly electrostatic) interactions determines the assembly state and morphology. a) Gold nanoparticles grafted with poly(4‐vinylpyridine) brushes in a fully swollen state (pH = 3.1).^[^ ^106^ ^]^ After triggered solvophilic‐to‐solvophobic transitions, different scenarios may occur. (b_1_) individual gold nanoparticles with collapsed poly(4‐vinylpyridine) shells (pH = 4.4).^[^ ^106^ ^]^ Electron micrographs in (a) and (b_1_) are reprinted with permission.^[^ ^106^ ^]^ Copyright 2008, American Chemical Society. (b_2_) Dimers of gold nanoparticles grafted with poly(*N*‐isopropylacrylamide)‐*block*‐PEG copolymers. Electron micrograph reprinted with permission.^[^ ^110^ ^]^ Copyright 2022, American Chemical Society. (b_3_) Chain‐assemblies of nanoparticles. Electron micrograph reprinted with permission.^[^ ^113^ ^]^ Copyright 2013, John Wiley and Sons. And (b_4_) compact, globular assemblies. Electron micrograph reprinted with permission.^[^ ^105^ ^]^ Copyright 2016, Elsevier.

In the first scenario, initially, a solvent‐swollen polymer‐brush layer serves to stabilize the individual core−shell‐type gold nanoparticle building blocks. Starting from there, this stabilizing layer can be transformed into a collapsed brush layer by changing the solvency conditions from good to poor, which can be realized by the addition of a non‐solvent, or by temperature‐triggered,^[^ ^6^, ^104^ ^]^ light‐triggered,^[^ ^105^ ^]^ or pH‐triggered^[^ ^106^ ^]^ solvophilic‐to‐solvophobic transitions within the polymer brush. Next, different self‐assembly scenarios can follow this transition (**Figure** **8**). First, the core−shell‐type nanoparticles can remain stable even after the transition into a collapsed non‐swollen polymer layer, as has been observed, e.g., for gold/poly(*N*‐isopropylacrylamide) core−shell colloids upon brush collapse initiated by heating above the lower critical solution temperature of the polymer layer in aqueous solution,^[^ ^6^, ^70^, ^107^, ^108^, ^109^ ^]^ and as well for poly(4‐vinylpyridine)‐grafted gold nanoparticles after raising the solution pH values from 3.1 to 4.4.^[^ ^106^ ^]^ Hence, even though the polymer layer faces a poor‐solvent environment, aggregation into supracolloids may not occur. For poly(*N*‐isopropylacrylamide)‐grafted gold nanoparticles, it was shown that assembly can be forced by adding electrolyte^[^ ^6^, ^107^, ^109^ ^]^ and also by adding excess polymer,^[^ ^108^ ^]^ which points to surface charges being the cause for the initially prevented aggregation, as elaborated in recent systematic work on that aspect.^[^ ^109^ ^]^ These surface charges result from initially present small molecules (typically citrate or charged surfactant molecules), which are introduced during the synthesis of nanoparticles in the ex situ strategy (vide supra) and typically not fully removed from the gold‐nanoparticle surface upon grafting with polymer ligands. Sometimes, surface charges are also deliberately introduced, e.g. by co‐adsorption of charged small‐molecule ligands together with the polymer ligands.^[^ ^110^ ^]^ When polymer‐grafted (gold) nanoparticles do assemble by solvophobic triggers, the assembly morphology may again be influenced by electrostatic forces. Specifically, systematic work^[^ ^16^ ^]^ has demonstrated the impact of the balance of (attractive) solvophobic interactions between the polymer layers and (repulsive) electrostatic forces between the colloidal particles. While solvophobic interactions may initiate nanoparticle association, the formation of compact assembly morphologies may be impeded by additionally present electrostatic repulsive interactions between constituent nanoparticles, leading to extended chain‐like assemblies.^[^ ^16^, ^111^, ^112^ ^]^ If this balance is influenced such that solvophobic interactions dominate while electrostatic repulsion plays a less important role, compact, globular assembly morphologies are obtained. This explanation is consistent with observed^[^ ^16^ ^]^ transitions from chain‐like assembly structures to globular assemblies in aggregating gold/polystyrene core−shell‐type nanoparticles by electrolyte additions (resulting in charge screening) and upon increasing the chain length of surface‐grafted polymers (resulting in thicker polymer shells and hence increased separation between the gold surfaces of associating gold nanoparticles). Chain assemblies of core−shell‐type nanoparticles are also observed in other cases where surface charges play a role, like for example in gold nanoparticles coated with poly{[2‐(dimethylamino)ethyl] methacrylate} brushes, where the brushes were additionally complexed by citrate.^[^ ^113^ ^]^ Here gradual pH decrease lowered the absolute value of negative zeta‐potentials (resulting from citrate), and the first assembled structures that were formed displayed chain‐like morphology.^[^ ^113^ ^]^ In another recent work,^[^ ^110^ ^]^ surface charges were modulated by co‐adsorption of different charged small molecules (mercapto‐*N,N,N*‐trimethylhexan‐1‐aminium bromide, cysteamine, and 3‐mercaptopropionic acid) in addition to poly(*N*‐isopropylacrylamide)‐*block*‐poly(ethylene glycol) diblock copolymer ligands, and surface‐charge modulation via this co‐adsorption and/or electrolyte additions enabled the controllable formation of nanoparticle chain assemblies with distinct dimensions (from dimers to oligomers and longer chains) and also sheet‐like assembly morphologies.^[^ ^110^ ^]^ Attenuation of the clustering at the dimer stage by fine‐tuning the electrostatic repulsive interaction was also achieved in a different system involving physisorbed polystyrene‐*block*‐poly(acrylic acid) diblock copolymer ligands at gold nanoparticles.^[^ ^114^ ^]^ The discussed influence of the balance between attractive solvophobic interactions and repulsive electrostatic interactions on the assembly state of polymer‐grafted gold nanoparticles is summarized in Figure 8.

In cases where electrostatic interactions are less important, like in solvent environments where ion pairs are not separated, globular assembly morphologies are typically observed upon solvophobicity‐induced nanoparticle agglomeration.^[^ ^105^ ^]^ The described examples illustrate how balancing attractive solvophobic interactions and repulsive electrostatic interactions can provide a strategy to control the architecture of aggregate structures comprising polymer‐grafted gold nanoparticles. A similar balance may also be established between solvophobic interactions and repulsive interactions that are of steric origin: For gold nanoparticles (42 nm) coated with mixed PEG/poly(methyl methacrylate) brushes, attenuation of the cluster formation process mostly at the dimer stage is observed^[^ ^115^ ^]^ under poor solvency conditions for poly(methyl methacrylate), but good solvency conditions for PEG (i.e., in aqueous solution). This observation was rationalized by solvophobic interactions between the poly(methyl methacrylate) part resulting in attraction between nanoparticles. It is speculated that upon the dimer formation process, solvent‐swollen PEG chains would be depleted from the dimer junction, thereby accumulating in the non‐contacting regions, resulting in steric stabilization of the formed dimers. To what extent electrostatic interactions contribute to the attenuation of the solvophobicity‐induced aggregation process at the dimer stage was however not investigated in this work.^[^ ^115^ ^]^

The second scenario: directed nanoparticle linking via specific interactions promoted by the polymer layer, is sometimes branded as the “brick and mortar” approach. Other than in the previously discussed case, the polymer “mortar” provides a more robust way of connecting the nanoparticles, which leads to a reduced dependence of the assembly state on changes in the solvent environment (solvent quality and dilution). Here, the incorporation of two or more gold‐affine groups, which can be distributed along the main chain,^[^ ^116^, ^117^ ^]^ at the chain ends of linear polymers (telechelics),^[^ ^117^, ^118^ ^]^ or chain ends of hyperbranched polymers^[^ ^119^, ^120^ ^]^ was used to make these polymers to nanoparticle cross‐linkers. Alternatively, a “brick‐and‐mortar” assembly of gold nanoparticles can be achieved by incorporating complementary functional groups into polymer ligands, which can promote nanoparticle cross‐linking via hydrogen‐bond interactions,^[^ ^121^ ^]^ and via photocrosslinking of coumarin‐moieties.^[^ ^122^ ^]^ The latter also enables the triggered dis‐assembly via [2+2]‐cyclo‐reversion upon illumination at a shorter wavelength.

Such materials made from cross‐linked gold nanoparticles enable a range of sensing modalities, where the signal transduction is based on distance‐dependent alteration of physical properties during the sensing event. For example, colorimetric humidity sensing in thin films composed of gold nanoparticles cross‐linked by telechelic PEG linkers has been achieved, where water vapor swells the polymer linker and increases gold interparticle spacings, resulting in a blue‐shift of the plasmon resonance frequency.^[^ ^123^ ^]^

### 3.2 Controlled Aggregation of Polymer‐Grafted Anisotropic Gold Nanoparticles

Shaped nanoparticles provide distinct surface sites, which can give rise to directional interactions during nanoparticle self‐assembly. Controlling and engineering such directional interactions is especially important for highly anisotropic gold nanoparticles, such as gold nanorods. Gold nanorods support two distinct plasmon modes, a transversal mode and a longitudinal mode that is red‐shifted with respect to the former and typically dominates the extinction spectrum. The longitudinal plasmon mode can be localized in the tissue‐transparency window of the NIR region, making gold nanorods and their assemblies promising candidates for in‐vivo applications, such as hyperthermia‐based tumor eradication.^[^ ^124^ ^]^ Depending on the geometry of self‐assembled structures, couplings to either of these modes (or both modes at the same time) can occur, which makes the properties of assembled structures strongly dependent on the assembly geometry. This is also true for the positioning of other functional entities within such nanoparticle assemblies, such as SERS markers^[^ ^125^ ^]^ or dyes.^[^ ^126^ ^]^ Thus, strategies for achieving the desired control at the supracolloidal scale are in great demand.

**Figure 9:**
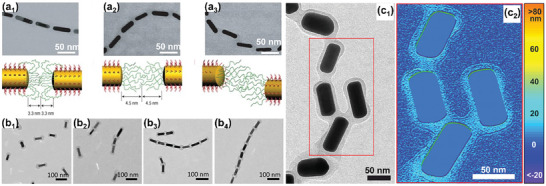
a) Self‐assembly phenomenology for polystyrene‐grafted gold nanorods in DMF/water mixtures (poor solvency conditions for polystyrene). Particle spacings increase with increasing water content: (a_1_) 10% wt. Water; (a_2_) 15% wt. Water; (a_3_) 20% wt. Water. Scale bars are 50 nm. Adapted with permission.^[^ ^129^ ^]^ Copyright 2007, Springer Nature. b) Observed structures after grafting gold nanorods with thiol‐terminated polystyrene at low target grafting density, stabilization of the structures with amphiphilic polystyrene‐*block*‐PEG block copolymers, and annealing at 90 °C in DMF/water (9% vol. water). Structures observed for different annealing times: (b_1_) 0.5 min; (b_2_) 1.0 min; (b_3_) 1.5 min; (b_4_) 2.5 min. Adapted with permission.^[^ ^131^ ^]^ Copyright 2023, American Chemical Society. c) Preferred tip‐to‐tip self‐assembly of gold nanorods uniformly coated with a polystyrene brush layer. (c_1_) bright‐field TEM image; (c_2_) polymer height map determined by energy‐filtered TEM. Adapted,^[^ ^62^ ^]^ Creative Commons under CC‐BY‐NC‐ND 4.0.

One rationale for exerting control over the self‐assembly process of the mentioned gold nanorods is site‐selective functionalization with organic ligands that subsequently promote inter nanoparticle binding. This was suggested as the mechanism of gold‐nanorod assembly triggered by adding small molecules (α,ω‐dithiols^[^ ^127^ ^]^ or thiolated carboxylic acids),^[^ ^128^ ^]^ which resulted in tip‐to‐tip assembly of nanorods. Similar assembly phenomenology was observed after the functionalization of gold nanorods with thiol‐end functional polystyrene ligands under poor solvency conditions for the polystyrene.^[^ ^129^, ^130^ ^]^ Also here, the observed tip‐to‐tip preference in the self‐assembly was attributed to gold‐nanorod functionalization exclusively at the tip sites, although this site‐selective functionalization was not directly proven (**Figure** **9**a). Another work^[^ ^131^ ^]^ presented a strategy that involved gold‐nanorod functionalization with thiol‐functional polystyrene ligands at low target grafting density, stabilization with amphiphilic polystyrene‐*block*‐PEG block copolymers, and annealing at elevated temperatures (90 °C) in DMF/water mixtures (poor solvency conditions for polystyrene). Under these conditions, surface de‐wetting occurred, which led to pinned‐micelle formation at the gold‐nanorod tips (see Figure 9b). In these pinned micelle systems, further minimization of the surface free energy during prolonged annealing resulted in the fusion of pinned micelles by particle assembly, which therefore created tip‐to‐tip assemblies of gold nanorods in high yield.

However, even without site‐selective pre‐functionalization, the distinct sites of gold nanorods can be discriminated. This is due to the effect of surface charges, which result from the surfactant (typically CTAB or CTAC, vide supra) used in the nanorod synthesis. These surface charges in combination with the distinct surface curvatures at the gold nanorod tips versus the sides result in distinct site‐specific interaction potentials which favor the tip‐to‐tip approach, as revealed by in situ liquid phase transmission electron microscopy.^[^ ^132^ ^]^ Surface charges can be maintained in gold nanorods, even after functionalization with non‐charged polystyrene ligands in polar organic solvents or mixtures of such solvents with water (due to incomplete substitution of parts of the surfactant molecules).^[^ ^133^ ^]^ Therefore, similar to the assembly mechanisms described above for spherical polymer‐grafted gold nanoparticles (Section 3.1), the superposition of attractive inter‐nanorod interactions (typically solvophobic interactions) on one hand, and repulsive electrostatic interactions, on the other hand, should be considered to arrive at a full picture of the self‐assembly mechanisms of polymer‐grafted gold nanorods in colloidal solution. We recently showed that polystyrene‐grafted gold nanorods assemble preferably in a tip‐to‐tip manner under poor solvency conditions for polystyrene, even when polystyrene is not selectively localized only at the nanorod tips (and depleted at the long sides), as revealed by locally resolved quantification of the polystyrene layer thickness via energy‐filtered transmission electron microscopy (Figure 9c).^[^ ^62^ ^]^ We demonstrated that remaining surface charges result in lower energy barriers for a tip‐to‐tip approach of the nanorods compared with the side‐to‐side approach, which makes the tip‐to‐tip assembly the kinetically favored assembly mode in this system.^[^ ^134^ ^]^

### 3.3 Co‐Assembly with Gold Nanoparticles

**Figure 10:**
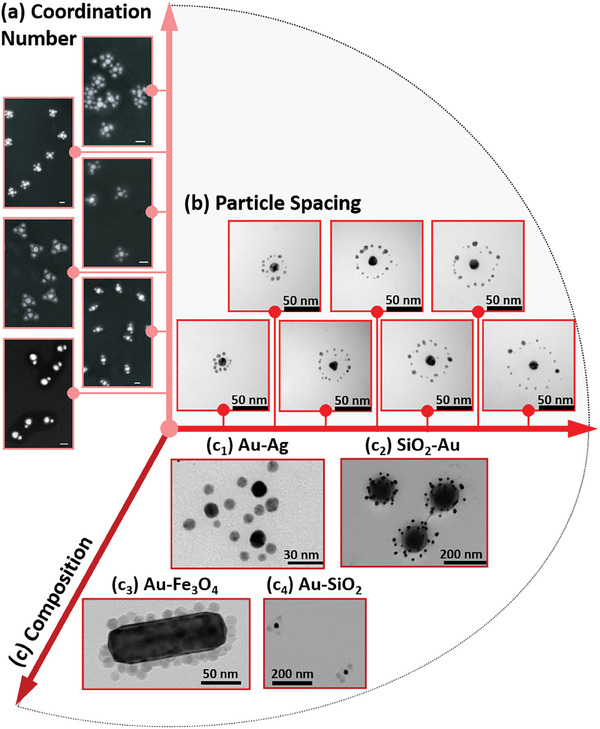
Structural variety of accessible planet−satellite‐type supracolloids, where polymers play the key role in directing the assembly. The following dimensions of supracolloidal architecture can be controlled: a) Coordination number.^[^ ^141^ ^]^ Scale bars are 50 nm. Reprinted with permission from AAAS. b) Particle spacing. Images adapted with permission.^[^ ^149^ ^]^ Copyright 2014, John Wiley and Sons. c) Composition, meaning the combination of gold nanoparticles with other kinds of nanoparticles. (c_1_) Image adapted with permission.^[^ ^151^ ^]^ Copyright 2016, American Chemical Society. (c_2_) Image adapted with permission.^[^ ^146^ ^]^ Copyright 2017, John Wiley and Sons. (c_3_) Image adapted.^[^ ^153^ ^]^ Creative Commons under CC BY 4.0. (c_4_) Image adapted with permission.^[^ ^154^ ^]^ Copyright 2022, John Wiley and Sons.

By joining together colloidal building blocks with different qualities (like chemical composition, dimension, and shape), the parameter space for supracolloidal structures widens tremendously. If such systems display some order rather than random particle arrangement, the individual nanoparticle building blocks have a clear spatial relationship with each other, and a structural hierarchy can emerge. Assemblies comprising different types of nanoparticles separated radially in two different layers provide an example.^[^ ^135^ ^]^ Another example is given by well‐defined “satellite” structures, in which another kind of nanoparticle is combined with a different “core” nanoparticle.^[^ ^136^ ^]^ In such and comparable systems, composition and structure determine the material properties. Synergistic effects can be exerted and also new emergent functional properties can be obtained: Interparticle coupling can enhance functional properties compared with the single particle level, for example, refractive‐index sensitivity,^[^ ^137^ ^]^ or can give rise to new phenomena, e.g., optical magnetism.^[^ ^138^, ^139^ ^]^ An overview of accessible satellite structures and structural parameters that can be controlled with the aid of grafted polymer ligands is displayed in **Figure** **10**. The polymer‐based synthetic strategies to access these supracolloids are discussed in the following, and their importance for controlling functional properties will be emphasized.

The number of constituent nanoparticles in supracolloidal structures is one key parameter that developing assembly strategies aim to control. The fundamental challenge associated with this goal is to incorporate attractive interparticle interactions that drive the association of individual nanoparticles, while also ensuring that this association is limited at a defined stage of the cluster formation process. The reward for tackling this challenge is structural precision mirroring the defined stoichiometry and geometry of molecules. Hence, the term “colloidal molecules” (or “plasmonic molecules” in the case of constituent metal nanoparticles) was hatched to describe these supracolloidal entities. The first steps toward their synthesis targeted (AB‐type) heterodimers of two distinct gold nanoparticles.^[^ ^140^ ^]^ These gold nanoparticles were sterically stabilized with PEG brushes before thiolated small molecules (mercapto‐dodecanoic acid and 11‐mercaptoundecyl‐trimethylammonium bromide) were adsorbed at their surface to introduce complementary charges at the distinct gold nanoparticles. Attractive electrostatic interactions between both kinds of nanoparticles initiated their assembly, while the resulting assembly state was controlled by the density of the PEG brush layer, which provided steric stabilization and hence prevented mere aggregation/precipitation of these oppositely charged colloids and also limited the clustering. Depending on the brush density, the mentioned heterodimers (and also linear chains with alternating sequences of the constituent nanoparticles) could be realized. More recently, the concept of self‐limiting nanoparticle bonding was developed as a synthesis paradigm for controlling the number of B‐type nanoparticles in AB*_n_*‐type colloidal molecules with impressively small dispersity (Figure 10a).^[^ ^141^ ^]^ This concept involves specific complementary interactions between polymer ligands at the two distinct nanoparticles. In the first realization, salt‐bridge formation between distinct gold nanoparticles served as the attractive interaction to promote colloidal‐molecule formation. For that, two different thiol‐terminated diblock terpolymers were used to functionalize gold nanoparticles with distinct sizes. These polymers both comprised nanoparticle‐remote PEG blocks, while the inner block was a random copolymer of styrene with poly(acrylic acid) in one case and of styrene with poly(*N,N*‐dimethylaminoethyl methacrylate) in the other case. The amount of acid/base functionality at the nanoparticles varies with nanoparticle dimension and grafting density, but can also be controlled independently from these parameters by adjusting the co‐monomer ratio in the random copolymer blocks comprising acid/base functionalities, and by the chain length of this block. Nanoparticle clustering ceased once all acid/base functionalities had reacted during their neutralization (i.e., salt‐bridge formation). Thus, the cluster stoichiometry (parameter *n* in AB*_n_*‐type colloidal molecules) could be controlled by adjusting the ratio of acid/base functionalities in the grafted polymer species.^[^ ^141^ ^]^ If the acid functionalities are derived via photocleavage of a precursor, irradiation time can be used as a handle to adjust this ratio and thus cluster stoichiometry.^[^ ^142^ ^]^ A related work^[^ ^143^ ^]^ demonstrated that hydrogen‐bond interactions between random copolymers of polystyrene/poly(4‐hydroxysytrene) (hydrogen bond donor) and PEG (hydrogen bond acceptor) as ligands on two distinct gold nanoparticles can also serve as the key attractive interactions in forming colloidal plasmonic molecules. As different solvent molecules also feature varying degrees of hydrogen‐bond donor/acceptor capability, there is competition between these solvent molecules and the two distinct polymer ligands. Colloidal molecule formation can be fully suppressed in some solvents (THF in this case), and initiated upon changing the solvent environment (by adding chloroform to THF solution in the case of this work).^[^ ^143^ ^]^ This provides the unique possibility to control the coordination number in colloidal molecules by keeping the same constituent nanoparticles and varying only the solvent composition. Halogen‐bond interactions between different copolymers of styrene with halogen‐bond donors (4‐bromostyrene, 4‐chlorostyrene, and 2‐(perfluorohexyl) ethyl methacrylate were studied) on one hand and halogen‐bond acceptors on the other hand (2‐(dimethylamino) ethyl methacrylate) have also been used^[^ ^144^ ^]^ to form colloidal plasmonic molecules. These interactions were found to allow reversible assembly/disassembly of this type of well‐defined supracolloidal clusters. The presented examples^[^ ^141^, ^143^, ^144^ ^]^ demonstrate the feasibility of controlling *n* in AB*_n_*‐type “colloidal molecules” up to moderate *n* values (≈7), which also mirrors the small molecule paradigm. For larger values of *n*, their exact control remains to be achieved, but the average value of *n* can be controlled (with some dispersion) by preventing saturation of the A‐type nanoparticles with B‐type nanoparticles, i.e., adjusting the number of B‐type nanoparticles in the assembly process.^[^ ^145^, ^146^ ^]^ Very recently, a strategy to control also the configuration of plasmonic molecules after deposition on a substrate was reported.^[^ ^147^ ^]^ Oftentimes, such nanoparticle clusters adopt a planarized structure upon deposition on a substrate and solvent evaporation, due to the structural plasticity of the system which leads to this re‐configuration during the drying process. It was shown that sonication treatment of polymer‐linked colloidal molecules can decrease interparticle distances and also result in a maintained 3D structure of the plasmonic molecules on a substrate. As an example, for AB_4_‐type supracolloids D_4h_‐symmetric structures result after deposition without prior sonication treatment, whereas such pre‐treatment gives rise to preserved T_d_‐symmetric structures also after drying on a surface.

As coupling interactions between plasmonic nanoparticles and also energy‐transfer processes that occur concomitant with the plasmon excitation are strongly distance‐dependent (such as plasmonic heating of the environment or interactions with other photo‐excited states such as excitons etc.),^[^ ^148^ ^]^ control over interparticle spacings in supracolloidal structures is of utmost importance. Synthetically, this parameter can be controlled by employing well‐defined (e.g., RAFT‐synthesized) star polymers with defined molar masses and gold‐affine branch termini as nanoparticle linker and spacer in planet−satellite‐type clusters (Figure 10b).^[^ ^149^ ^]^ In these systems, particle spacings are determined by the arm length of the star polymer linker, or linear bifunctional (telechelic) polymeric linker (which formally is equal to a two‐arm star polymer).^[^ ^66^ ^]^ The synthetic approach is based on a modular procedure, where the polymer linker molecules are first adsorbed at the surface of the “planet” particle. Because not all end monomers of these star polymers anchor to the nanoparticle surface as a result of their crowding and the associated high free‐energy barriers for diffusing an end monomer to the “planet”‐particle surface, some end monomers remain accessible within the polymer layer.^[^ ^51^ ^]^ The position of the end‐monomer distribution roughly determines the position of grabbed “satellite”‐type particles in the subsequent assembly process, while the magnitude of this end‐monomer distribution – which can be varied by changing star polymer arm number at conserved arm length – is correlated with the number of “satellite” particles that can become attached.^[^ ^66^ ^]^ If the assembly involves like‐charged “planet” and “satellite” nanoparticles in aqueous environments, their approach is hampered due to electrostatic repulsion. However, the addition of electrolyte (for charge screening) together with the use of a weakly physisorbed, neutral surfactant (to prevent aggregation of nanoparticles as a result of electrolyte addition) can be used to access planet−satellite‐type clusters also in such situations.^[^ ^150^ ^]^

Finally, the chemistry of nanoparticles constituting hierarchical co‐assemblies with gold nanoparticles can vary. Figure 10c illustrates four examples: Figure 10c_1_) Gold‐planet−silver‐satellite nanostructures,^[^ ^151^ ^]^ which display, e.g., synergistic effects in catalysis, where the catalytic activity of the silver nanoparticles is raised by plasmonic heating upon excitation of the nearby gold nanoparticles.^[^ ^152^ ^]^ Figure 10c_2_) Thermoresponsive (poly(*N*‐isopropylacrylamide)‐linked) silica‐planet−gold‐satellite nanostructures,^[^ ^146^ ^]^ in which the arrangement of plasmonic nanoparticles around a dielectric core can give rise to optical magnetism.^[^ ^138^, ^139^ ^]^ Figure 10c_3_) Gold‐nanorod‐planet−iron‐oxide‐satellite nanostructures, which can be aligned in response to a static or dynamic external magnetic field.^[^ ^153^ ^]^ This alignment reveals the optical anisotropy of the gold nanorods at the macroscopic level: The scattering and absorbance of the colloidal solution are now dependent on gold nanorod orientation in relation to the direction of light propagation. It is thereby possible to visualize and track external magnetic fields Figure 10c_4_) Gold‐planet−silica‐satellite nanostructures with defined satellite number and placement as well‐defined supracolloidal building blocks.^[^ ^154^ ^]^ Other interesting effects that can be realized based on the combination of distinct nanomaterial properties include plasmon−exciton coupling by arranging quantum‐dot satellites around gold nanoparticles^[^ ^155^ ^]^ or vice versa.^[^ ^156^ ^]^

While some possibilities that open up due to the combination of distinct nanomaterial properties have been described above, one may ask: How does the structural identity of supracolloidal co‐assemblies – in particular particle stoichiometry and interparticle spacing – influence emerging effects/application scenarios? Oftentimes, a high number of strong coupling interactions and hence many interacting particles with short spacings are sought. This is the case for, e.g., plasmon−plasmon coupling interactions that create local “hot spots” with high electric field enhancement. Thus, applications that are near‐field promoted, such as SERS, provide an illustrative example.^[^ ^157^, ^158^ ^]^ Here, the achieved SERS enhancement increases by decreasing the length of the polymeric linker^[^ ^157^ ^]^ and is proportional to the number of attached satellites, i.e., the number of hot spots that are being created.^[^ ^145^ ^]^ However, for some phenomena like luminescence enhancement and photocatalytic rate enhancement observed when placing an emitter/photocatalyst in an electric hot spot created by interacting plasmonic nanoparticles, there is a certain distance (typically of the order 10^1^ nm) for maximized luminescence enhancement^[^ ^159^ ^]^ / photocatalytic rate enhancement,^[^ ^160^ ^]^ and reduced particle separations will result in the loss of the enhancement or even reduce the magnitude of the effect compared with the uncoupled reference system. Of course, the respective particle spacing that leads to a maximized magnitude of the desired effect depends on the individual characteristics of the systems. Thus, interparticle spacings in supracolloidal co‐assemblies of gold nanoparticles are critical to exert control over the mentioned functional properties of such systems.

The selected illustrative examples already reveal the rich variety of static functional supracolloidal systems that can be realized and tailored to purpose, based on synthetic polymers as key components in guiding the assembly structure. The scope of such functional supracolloidal systems can be further widened by taking the step to dynamic systems. The possibilities that are offered by such dynamic supracolloidal systems are explored in the following section.

## 4 Toward Dynamic Gold‐Containing Supracolloidal Systems Enabled by Grafted Polymers

As discussed in the previous section, the switching between assembled/disassembled states of supracolloids have been achieved based on a range of external triggers such as pH, temperature, or light. These systems already fulfill the definition of reconfigurable colloidal assembly, which has been given as such:^[^ ^161^ ^]^ *“It involves reversible, back and forth transitions between two different equilibrium states of a colloidal system by the manipulation in time of thermodynamic variables or colloidal properties*.” The dynamic supracolloids that we will review in this section fall into this broad definition. At the same time, dynamic supracolloids represent a special case of a re‐configurable colloidal system. Thus, we can give the following definition to characterize dynamic supracolloids: *Supracolloids with dynamic behavior feature externally triggered, reversible changes in structure and properties over time, while the overall assembly state is preserved*. These changes can be mediated by reconfigurable polymeric linkers that – depending on their chemical nature – process distinct external stimuli by a change in their conformation. These conformational changes are then translated to the supracolloidal scale. While examples of such supracolloidal systems enabled by biomacromolecular linkers (especially DNA) also exist,^[^ ^162^, ^163^ ^]^ we focus here on systems based on synthetic polymer linkers. Mechanisms and realizations of such dynamic supracolloids are given below. We will categorize these systems according to the type of response mediated by the polymer component: The polymer component may support gradual changes in response to external parameters, which can be discriminated from critical phenomena.

**Figure 11:**
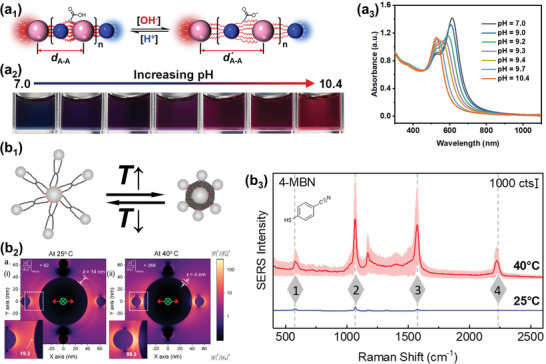
(a_1_) Dynamic supracolloidal chain assemblies composed of gold nanoparticles with distinct dimensions arranged in an alternating manner. (a_2_) Colloidal solution color in response to changing pH and (a_3_) associated extinction spectra. Adapted with permission.^[^ ^166^ ^]^ Copyright 2024, John Wiley and Sons. (b_1_) Dynamic supracolloidal planet‐satellite‐type structures composed of gold nanoparticles of distinct dimensions [note: In reality, nanoparticles are linked by several star polymer molecules, which is omitted here for clear display]; (b_2_) reversible switching of electromagnetic hot‐spot sites by actuating interparticle gap distances; and (b_3_) SERS signal intensity after adsorption of 4‐mercaptobenzonitrile. Adapted,^[^ ^169^ ^]^ Creative Commons under CC BY‐NC 3.0.

The former mechanism (gradual changes in polymer conformation) was established for systems that were coined “spring”‐like assemblies. One of the first realizations is a nanoscale thermometer, which was established by CdTe quantum dots assembled as “satellite” particles around a gold nanoparticle core by PEG linkers. In these systems, temperature‐dependent gradual swelling of the PEG linker resulted in an alteration of the gold−quantum‐dot spacing, with a concomitant modulation of the photoluminescence intensity of the quantum dots.^[^ ^164^ ^]^ Thus, it is in principle possible to determine the effective local temperature in a small confined space around a plasmonic nanoparticle, by the transducing property of the polymeric linker entity. Such and similar possibilities enable the creation of “messenger materials”, which inform about nanoscopic environments by easily processable signals (such as luminescence or color change).^[^ ^165^ ^]^ In another very recent example, pH served as the external trigger to affect gap‐spacing modulation in assemblies of gold nanoparticles: Supracolloidal analogs of alternating co‐polymers, in which PEG‐grafted gold nanoparticles with co‐adsorbed (11‐mercaptoundecyl)‐*N,N,N*‐trimethylammonium bromide (to impart positive surface charge) in one case, and grafted poly(acrylic acid) in the other case formed chain‐like assemblies with alternating expression of (distinctly sized) gold nanoparticles under appropriately chosen ionic strength of the solution.^[^ ^166^ ^]^ Here, the interparticle spacing could be adjusted by changing the solution pH value, leading to different protonation states and thus conformations of the poly(acrylic acid) component (**Figure** **11**a). Resulting changes in interparticle spacing were evidenced by SEM imaging after drying, but also indirectly concluded from extinction spectroscopy that revealed distinct solution colors resulting from the distance‐dependent modulation of plasmon coupling.^[^ ^166^ ^]^

The mentioned critical phenomena supported by polymer linkers could be used to realize switchable supracolloids. Examples of switchable supracolloids include planet−satellite‐type nanostructures with polymer linkers featuring a lower critical solution temperature (LCST). As discussed in Section 3.1 for core−shell‐type nanoparticles with LCST‐type polymer brush ligands, heating‐induced shell collapse may result in aggregation or stable individual particles with collapsed shells, depending on the effect of remaining surface charges. Similarly, for particular cases, heating‐induced polymer‐linker collapse can result in reversible contraction and expansion of the distance of satellite particles from the core of the structure, without aggregation.^[^ ^150^ ^]^ For supracolloidal clusters where aggregation would occur after heating homopolymer‐type linkers above their LCST, this aggregation can be circumvented by employing block‐copolymers, where inner temperature‐responsive poly(*N*‐isopropylacrylamide) blocks (of a linking star polymer) are combined with stabilizing outer poly(*N,N*‐dimethylacrylamide) blocks that remain solvent‐swollen in the temperature range of interest.^[^ ^167^ ^]^ Using this strategy, actuation of planet−satellite distances can also be achieved for cluster systems that would otherwise become colloidally unstable. Other possibilities offered by the block structure of the polymer linker include the incorporation of both LCST‐ and UCST‐behavior, which was realized based on poly[di(ethylene glycol) ethyl ether acrylate‐co‐oligoethylene glycol acrylate]‐*block*‐poly[*N*‐(3‐(dimethylamino)propyl) methacrylamide] terpolymers.^[^ ^168^ ^]^ In this system, contraction of the planet−satellite spacing could be achieved by both heating and cooling, while expanded structures existed in a convenient temperature range between ≈25 and 45 °C. Reversible switching between an expanded and contracted supracolloidal state in such systems gives rise to plasmon‐based thermochromism.^[^ ^150^, ^168^ ^]^

Besides, such size switching could be advantageous in situations when loading of interparticle gap regions (e.g., with analyte molecules) requires an expanded state, while subsequent steps (e.g., detection of analyte molecules) require strong plasmon‐plasmon coupling and hence a contracted state. In recent work,^[^ ^169^ ^]^ we demonstrated this possibility (Figure 11b). Comparably large gold nanoparticles were assembled into planet−satellite structures (80 nm diameter for the planet particle and 16 nm for the satellites) by a 4‐arm poly(*N*‐isopropylacrylamide) star polymer linker synthesized by RAFT polymerization. These supracolloids could be reversibly contracted and expanded by heating/cooling above/below the polymer's LCST. This allowed the adsorption of a model analyte (4‐mercaptobenzoic acid) into the hot spots in the expanded state, and its SERS‐based detection in the strongly coupled contracted state after heating to 40 °C. A functional property can also be switched off rather than enhanced after applying an external trigger, as was the case for the catalytic activity gold satellites (attached to a metal‐oxide core) upon the heating‐induced collapse of polymer brushes attached to those satellite particles (impeding access to the gold surface).^[^ ^170^ ^]^ Another example, although not a brush‐based system, is given by switchable optical magnetism, realized by actuating differently sized gold nanoparticles or gold/silver nanoparticles co‐assembled at a poly(*N*‐isopropylacrylamide) hydrogel particle.^[^ ^171^ ^]^

## 5 Conclusion and Outlook

Surface functionalization with polymer ligands is a powerful and extremely versatile approach for controlling the properties of colloidal gold in solution. Structural properties of such systems can be targeted and engineered to the purpose, including also the possibility of breaking the symmetry of spherical gold nanoparticles by introducing heterogeneities in the polymer coating layer. The huge versatility polymer ligands offer in terms of chemical composition – further expanded by the possibility of combining different polymers in mixed brush systems – allows to equip these particles with desired functional properties. The versatility is mirrored in the manifold of solvent environments in which these systems can be applied, covering a wide range of solvent types (water as well as organic solvents and solvent mixtures), ionic strengths, and temperatures – an important advantage over comparable systems based on, e.g., biomacromolecular surface ligands.

The step from single hybrid (polymer/gold) nanoparticles to supracolloidal systems further widens the scope of such nanomaterials by the possibility of controlling particle arrangement by appropriate choice of polymer coating. This structural control at the supracolloidal scale allows for a combination of different nanoparticles in one well‐defined nanoarchitecture; or to tailor interparticle coupling interactions between similar nanoparticles (such as plasmon−plasmon interactions) and interactions between different kinds of nanoparticles (such as plasmon−exciton interactions). Synthesis strategies have been developed to precisely control the important parameters in static supracolloidal solution assemblies: control over coordination number, interparticle spacing, and combination of gold and different inorganic nanoparticles have all been realized based on polymer‐mediated supracolloidal assembly approaches. Given the great success in this domain, especially in terms of the appreciable yield of supracolloids and the achieved structural precision, it would be tempting to explore the assembly of such supracolloids into bulk materials, as has been recently achieved for colloidal building blocks.^[^ ^172^, ^173^ ^]^ In addition to such solution‐based strategies, the template‐assisted assembly of supracolloidal structures^[^ ^174^ ^]^ also provides interesting prospects for the formation of optical (meta‐)materials, which are not yet fully explored. Parallel to these advances in assembling supracolloidal structures with polymer‐grafted gold nanoparticles, their controlled dis‐assembly and the recovery of the distinct constituent nanoparticles is an important goal from an applied standpoint while uncovering the underlying mechanisms of supracolloid dis‐assembly is also an appealing target for fundamental research. It can be anticipated that the conformational flexibility of the polymeric linking entity and the resulting responsivity to the environment can also be fruitfully used for establishing supracolloidal disassembly strategies.

The conformational flexibility of polymeric linkers employed in supracolloids also allows for control of the dynamic behavior of this system, which adds another dimension to these nanomaterials in comparison with static assembled structures. Recent developments in this regard have been covered in the final section of this review. In the future, these materials could feature, e.g., optical and catalytic properties that could be activated/deactivated on demand; or even adaptive behavior with regard to these properties, that would allow a different processing of external parameters depending on previous events. It can thus be expected that these realizations of dynamic hybrid polymer/gold supracolloidal structures only mark the beginning of the exploration of truly 4D‐programmed functional supracolloids.

## Conflict of Interest

The authors declare no conflict of interest.
